# Long-Lasting Defence Priming by β-Aminobutyric Acid in Tomato Is Marked by Genome-Wide Changes in DNA Methylation

**DOI:** 10.3389/fpls.2022.836326

**Published:** 2022-04-15

**Authors:** Marco Catoni, Raul Alvarez-Venegas, Dawn Worrall, Geoff Holroyd, Aarón Barraza, Estrella Luna, Jurriaan Ton, Michael R. Roberts

**Affiliations:** ^1^School of Bioscience, University of Birmingham, Birmingham, United Kingdom; ^2^Departamento de Ingeniería Genética, CINVESTAV-IPN, Unidad Irapuato, Guanajuato, Mexico; ^3^Lancaster Environment Centre, Lancaster University, Lancaster, United Kingdom; ^4^CONACYT-CIBNOR, Centro de Investigaciones Biológicas del Noroeste, La Paz, Mexico; ^5^School of Biosciences, Institute of Sustainable Food, University of Sheffield, Sheffield, United Kingdom

**Keywords:** priming, induced resistance (IR), biotic stress, hypomethylation, tomato, beta-aminobutyric acid, DNA methylation

## Abstract

Exposure of plants to stress conditions or to certain chemical elicitors can establish a primed state, whereby responses to future stress encounters are enhanced. Stress priming can be long-lasting and likely involves epigenetic regulation of stress-responsive gene expression. However, the molecular events underlying priming are not well understood. Here, we characterise epigenetic changes in tomato plants primed for pathogen resistance by treatment with β-aminobutyric acid (BABA). We used whole genome bisulphite sequencing to construct tomato methylomes from control plants and plants treated with BABA at the seedling stage, and a parallel transcriptome analysis to identify genes primed for the response to inoculation by the fungal pathogen, *Botrytis cinerea*. Genomes of plants treated with BABA showed a significant reduction in global cytosine methylation, especially in CHH sequence contexts. Analysis of differentially methylated regions (DMRs) revealed that CHH DMRs were almost exclusively hypomethylated and were enriched in gene promoters and in DNA transposons located in the chromosome arms. Genes overlapping CHH DMRs were enriched for a small number of stress response-related gene ontology terms. In addition, there was significant enrichment of DMRs in the promoters of genes that are differentially expressed in response to infection with *B. cinerea*. However, the majority of genes that demonstrated priming did not contain DMRs, and nor was the overall distribution of methylated cytosines in primed genes altered by BABA treatment. Hence, we conclude that whilst BABA treatment of tomato seedlings results in characteristic changes in genome-wide DNA methylation, CHH hypomethylation appears only to target a minority of genes showing primed responses to pathogen infection. Instead, methylation may confer priming via *in-trans* regulation, acting at a distance from defence genes, and/or by targeting a smaller group of regulatory genes controlling stress responses.

## Introduction

Induced resistance (IR) responses mounted by plants to improve their ability to withstand environmental stress are becoming increasingly recognised as potential targets for crop protection systems. The ability to enhance natural biotic stress resistance mechanisms could make an important contribution to integrated pest and disease management systems aimed at reducing reliance on synthetic pesticides in agriculture (Stenberg, 2017). In turn, by reducing chemical inputs whilst at the same time maximising outputs, integrated pest and disease management systems will play a critical role in future sustainable agricultural strategies (Pretty, 2018).

IR responses are triggered in the presence of biotic stress and are recognised by the subsequent increase in resistance both to the current challenge, and in many cases, to future challenges too (de Kesel et al., 2021). An area of significant interest in longer-lasting IR is the process known as priming. As well as immediate activation of inducible defences following stress, plants can also enhance future defences without long-term production of costly defensive metabolites and proteins. The establishment of priming creates a heightened state of alert in which defence responses can be triggered more rapidly and/or to a greater degree in response to a second stress encounter (Conrath et al., 2015; Mauch-Mani et al., 2017; Wilkinson et al., 2019). Because it does not involve constitutively elevated defence, priming optimises the trade-off between the costs and benefits of defence (van Hulten et al., 2006). In many cases, priming can be maintained for long periods, extending for the lifetime of the plant, and even in some instances, into the next generation (Luna et al., 2012; Rasmann et al., 2012; Slaughter et al., 2012). As well as natural infection by pests and diseases, the application of various chemical agents can also trigger immediate IR and longer-term defence priming responses. Such chemicals include endogenous plant hormones and other signalling molecules such as jasmonic acid (JA; Worrall et al., 2012), pipecolic acid (Bernsdorff et al., 2016) and C_6_ green leaf volatiles (Scala et al., 2013), biological molecules from non-plant sources, such as plant growth-promoting rhizobacteria (PGPR; Pieterse et al., 2014), and synthetic mimetics of biological signalling molecules, such as (*R*)-β-homoserine (Buswell et al., 2018).

The long-term nature of many priming responses raises questions as to the mechanism by which “memories” of stress are maintained in plants. A number of potential mechanisms have been identified as playing roles in priming (Conrath et al., 2015; Mauch-Mani et al., 2017). These mechanisms likely act over different time scales and are not mutually exclusive. Relatively short-term stress memory may be achieved via the increased production (possibly in an inactive form) of stress signalling proteins, such as pattern recognition receptors or protein kinases (Beckers et al., 2009), or the accumulation of conjugated, inactive phytohormones. More recently, a wider role for metabolism has been proposed, whereby “metabolic imprints” of stress responses can contribute to priming memory (Schwachtje et al., 2019). Stress memory can also be programmed epigenetically, at the level of gene expression, since packaging of DNA into chromatin controls access to transcription factors and the core transcriptional machinery. Chromatin structure can be altered in response to environmental experience through a variety of post-translational modifications of histone proteins, and histone modifications have been linked with priming and stress memory in several systems (Buzas, 2017; Lämke and Bäurle, 2017; He and Li, 2018). Similarly, the degree of DNA methylation at chromosomal loci containing genes is also linked with their expression, with higher levels of methylation associated with transcriptionally inactive heterochromatin and reductions in methylation associated with activation of gene expression (Zhang et al., 2018). Priming of environmentally regulated genes, including those involved in IR, has been suggested to be a consequence of stress-induced cytosine demethylation, particularly for genes located close to highly methylated chromosomal regions containing repetitive DNA sequences such as transposable elements (TEs; Yu et al., 2013; Wilkinson et al., 2019; Parker et al., 2021; Wang et al., 2021; Zhi and Chang, 2021).

One of the best-studied inducers of priming in plants is beta-aminobutyric acid (BABA). Identified only recently as a natural plant metabolite (Thevenet et al., 2017), exogenous application of BABA has long been known to prime resistance to a range of both biotic and abiotic stresses in many different plant species, including tomato (*Solanum lycopersicum*; Baccelli and Mauch-Mani, 2016; Cohen et al., 2016). Application of BABA to tomato, either by seed treatments, foliar sprays, or root drenches, provides improvements in broad spectrum resistance to diseases caused by biotrophic and necrotrophic bacterial, fungal and oomycete pathogens (Worrall et al., 2012; Cohen et al., 2016; Luna et al., 2016, 2020). Targets for BABA priming that result in enhanced defence in tomato mirror those seen in other plant species and include augmented transcriptional responses of defence genes (e.g., Worrall et al., 2012; Farahani et al., 2016), protective mechanisms against reactive oxygen species (Roylawar and Kamble, 2017) and changes in metabolic profiles (Luna et al., 2020). BABA generates long-lasting priming responses in tomato (spanning the lifetime of the plant) when applied to seeds or seedlings (Worrall et al., 2012; Wilkinson et al., 2018). Although transgenerational effects of BABA have not yet been reported in tomato, in the related Solanaceous species, potato (*Solanum tuberosum*), and in *Arabidopsis thaliana*, priming memory can be passed from BABA-treated plants to their offspring (Slaughter et al., 2012; Floryszak-Wieczorek et al., 2015; Meller et al., 2018; Kuźnicki et al., 2019).

In Arabidopsis, long-term within-generation priming by BABA depends on the activity of the H3K9 histone methyltransferase, *SUVH4/KRYPTONITE* (*KYP*), which affects chromatin structure both directly, via histone methylation, but also indirectly via impacts on CHG DNA methylation (Luna et al., 2014). Epigenetic mechanisms have also been studied in potato in an attempt to identify markers of long-term priming established following BABA treatment. Meller et al. (2018), found that methylation of histone H3 (H3K4me2) was enriched in the gene body of salicylic acid (SA)-dependent genes that were also primed for enhanced expression in response to infection by *Phytophthora infestans*. Subsequently, using the same experimental system, dynamic changes in DNA methylation were revealed following BABA treatment. BABA triggered rapid changes in genes encoding DNA methyltransferases and DNA glycosylases and led to an initial increase in global DNA methylation. However, over time, methylation gradually reduced to a level below controls, and hypomethylation at the promoter of a disease resistance gene, *R3a*, was transmitted to the next generation and correlated with primed transcriptional response to infection (Kuźnicki et al., 2019). Although evidence therefore exists for a role of DNA methylation in long-lasting priming of BABA-IR, whole genome methylation profiles have not been examined to date. Here, we employ whole genome bisulphite sequencing to profile changes in the methylome of tomato in response to BABA treatment at single base resolution.

## Materials and Methods

### Plant Growth and Beta-Aminobutyric Acid Treatments

Seeds of tomato (*Solanum lycopersicum* L., cv. Money Maker), were germinated in a peat-based compost mixture (Levingtons M3) and cultivated in a heated, passively ventilated glasshouse (min 18 ± 2°C, max 25 ± 3°C) with supplementary lighting (Osram Greenpower 600 W high pressure sodium lamps) to a minimum 250 ± 25 μmol.m^–2^.s^–1^ PAR at the canopy. A minimum 16-h photoperiod was maintained. Seven-day-old seedlings were root-drenched with a volume of 5 mM BABA equivalent to one tenth of the volume of the compost growing medium to achieve a final concentration of 0.5 mM BABA around the root system, as previously described (Luna et al., 2016). One week later, seedlings were gently removed from the compost, their root systems rinsed in water and then re-potted to new compost without BABA. Control plants were similarly treated but given a water root drench rather than BABA. After re-potting, plants were returned to the greenhouse.

### Whole Genome DNA Methylation Analysis

DNA from water and BABA-treated plants (21-days old plants, 7 days following the end of the BABA treatment period) was extracted using the DNeasy Plant Mini Kit (Qiagen) as per manufacturer’s instructions. Preparation of libraries and whole genome bisulphite sequencing (WGBS) were performed by GATC Biotech Ltd. using the Illumina HiSeq 4000 platform with 2 × 150 bp paired-end reads. Raw reads were trimmed using Trimmomatic (Bolger et al., 2014). High quality trimmed sequences were mapped against the tomato reference genome SL3.0^1^ using Bismark (Krueger and Andrews, 2011). The tomato chloroplast sequence (NC_007898.3) was used to estimate the bisulphite conversion rate. To account for non-converted DNA, we used the estimated conversion rate to apply a correction according to Catoni and Zabet (2021). Differentially methylated regions (DMRs) were identified between BABA-treated and mock-treated replicates using the R package *DMRcaller* (Catoni et al., 2018). We screened DMRs separately for the three cytosine methylation contexts (CG, CHG and CHH) with the function *computeDMRsReplicates*, using the “bins” method with a bin size of 200 nt, with 0.2 as minimum methylation difference, 2 as minimum cytosine count, 4 as minimum cytosine coverage and a minimum *p*-value of 0.05. Annotated genes and repeats from tomato reference genome annotation (ITAG3.2) were used to extract genomic features. The Repeat Masker annotation was used to define the class and the family of each TE. The overlap of DMRs and genomics features were calculated in R with the *GenomicRanges* package and compared to the overlap found using randomly generated regions (200 bp size) along the genome. Gene ontology (GO) term enrichment analysis was performed using the PANTHER classification system (Mi et al., 2020).

### Pathogen Infections

*Pseudomonas syringae* pv. *tomato* DC3000 (PstDC3000) luxCDABE-tagged strain (Fan et al., 2008) was used to inoculate tomato plants by dipping whole plants into the test bacterial solution (1 × 10^8^ cfu.ml^–1^, 10 mM MgCl_2_, 0.05% Silwet L-77) for 10 s. Mock-inoculated plants were treated with 10 mM Mg Cl_2_ (0.01% Silwet L-77) without bacteria and maintained under similar conditions. Six days after infection, three 1 cm^2^ leaf discs per plant were excised (each one from independent leaflets), rinsed and homogenised with 1 ml of 10 mM MgCl_2_, and plated in serial dilutions on King’s B media containing kanamycin and rifampicin (50 μg/mL each). The percentage of leaf damage on leaves exposed to the pathogen (total chlorotic + necrotic leaf area/total leaf area), was determined from three biological replicates with Fiji software (Schindelin et al., 2012). Inoculations of tomato leaves with *Botrytis cinerea* were performed as previously described (Worrall et al., 2012).

### Transcriptome Microarray Analysis

Seedlings were root drenched with BABA as described above. Ten days following re-potting to clean compost, detached leaves from control and BABA-treated plants were inoculated with droplets of germination medium either with *Botrytis* spores or without spores (mock inoculation). Five leaflets per leaf were inoculated with two droplets per leaflet. A total of six leaves per treatment per time point were harvested for RNA extraction and used to provide three independent biological replicates. Four leaflets per leaf were sampled by removing a 1 cm strip of leaf tissue including the two inoculation sites. Leaves from two independent plants were pooled for each RNA sample. Each RNA sample therefore contained a total of 16 inoculation sites from two plants. A total of 48 samples were collected, representing three replicates for each of the four time points for both the mock and *Botrytis* inoculations in each of the control and primed plant groups.

RNA was extracted essentially as described by Verwoerd et al. (1989), scaled up accordingly, and then purified using Qiagen RNeasy spin columns (Qiagen, United Kingdom). Microarray hybridisations were performed using the Affymetrix GeneChip^®^ Tomato Genome Array by the Nottingham Arabidopsis Stock Centre Affymetrix Service. Affymetrix array signals were normalised using the R package *rma*. The initial collection of 10,038 probe sets was filtered by removing the bottom tenth centile based on maximum signal intensity across all 48 samples and then by subsequent removal of the 20% of probe sets with the lowest standard deviation across all samples. This left 7767 probe sets for further analysis. Initial examination indicated that one sample (one of the BABA 6 h mock treatments) was compromised and it was therefore removed from further analyses. Differentially expressed genes were identified using “Bayesian Estimation of Temporal Regulation” (Aryee et al., 2009). Principal components analysis (PCA) and hierarchical clustering were implemented using the ClustVis web tool (Metsalu and Vilo, 2015). Functional classification of differentially expressed genes was performed using the ‘‘GO term enrichment analysis’’ tool at the Tomato Functional Genomics database^2^ using the Tomato Affymetrix Genome array probe set annotation as the target. Terms were searched against each of the “Process” and “Function” ontologies using a simulation method for *P*-value correction for multiple hypothesis testing.

## Results

### Beta-Aminobutyric Acid Root Drench Treatment Results in Genome-Wide Hypomethylation

**FIGURE 1:**
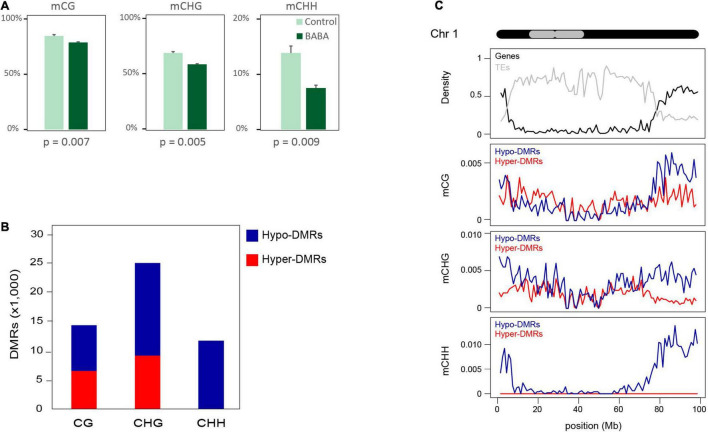
Global changes in DNA methylation patterns in response to BABA priming. **(A)** Global methylation levels (% of total cytosine bases) in control and BABA-primed plants. **(B)** Numbers (thousands) of DMRs in each cytosine context with increased (Hyper-DMRs) or decreased (Hypo-DMRs) methylation in BABA treated plants compared to controls. **(C)** Density of differentially methylated regions across chromosome 1 in each context (mCG, mCHG, and mCHH) separated into regions gaining (Hyper-DMRs; red lines) or losing (Hypo-DMRs; blue lines) methylation after BABA treatment. The chromosome structure is displayed in black at the top with the grey area marking heterochromatic regions (Zhong et al., 1998). Gene (black line) and TE (grey line) densities are displayed in the top track. Density was computed in a 1 Mb window along the chromosome.

We have previously shown that a BABA root drench treatment of 1-week-old tomato seedlings provides long-lasting priming of disease resistance (Luna et al., 2016; Wilkinson et al., 2018). To investigate whether epigenetic changes are associated with long-term priming, we performed whole genome bisulphite sequencing (WGBS). Following a root drench treatment of tomato seedlings, BABA-induced resistance was verified by a bacterial disease resistance assay on a subset of plants from each treatment. Compared with control plants, plants grown from BABA-treated seedlings displayed significantly reduced symptom development and bacterial population counts following inoculation with *Pseudomonas syringae* (Supplementary Figures 1A,B). The DNA from leaf tissues of each of three independent plants from each treatment was then extracted for WGBS, 14 days after root drenching. More than 20 M reads per sample were sequenced, with an average 9X coverage of the tomato genome (Supplementary Table 1). After quality control of sequencing output, we mapped the reads to the tomato reference genome and produced a DNA methylation profile at single cytosine resolution for each sequenced sample. The proportion of methylated cytosine observed in the mock-treated plants was consistent with previous reported methylation profiles of tomato (Zhong et al., 2013; Gouil and Baulcombe, 2016). However, the analysis of global methylation levels in BABA-treated plants revealed significant reductions in cytosine methylation in all three sequence contexts, estimated as approximatively 6, 10, and 6% of total cytosines, respectively, for CG, CHG and CHH contexts (Figure 1A). Considering this reduction in relation to the proportion of methylated genome for each cytosine context, hypomethylation was most pronounced for CHH, with the frequency of methylated CHH positions reduced by 38% in BABA-treated plants. In comparison, the reduction in CG and CHG methylation was only 6 and 12%, respectively.

### Hypomethylation Is Concentrated in the Chromosome Arms

We screened for differentially methylated regions (DMRs) separately for each cytosine sequence context and identified a total of 14,345 CG DMRs, 24,941 CHG DMRs, and 11,709 CHH DMRs. Whilst we found both hypo- and hypermethylated regions for CG and CHG contexts, for the CHH context, we observed that virtually all DMRs represented regions that were hypomethylated in BABA-treated plants (Figure 1B). When the positions of these DMRs were plotted alongside chromosome features for each of the 12 tomato chromosomes, there was a consistent trend for the majority of hypo-DMRs to be located in the gene-rich chromosome arms, while hyper-DMRs were more uniformly distributed along the chromosomes (Supplementary Figure 2). This pattern was noticeable for CG and CHG methylation but was especially striking for mCHH hypo-DMRs. As a representative example, a more detailed profile of distributions of DMRs and chromosomal features is shown for chromosome 1 in Figure 1C.

**FIGURE 2:**
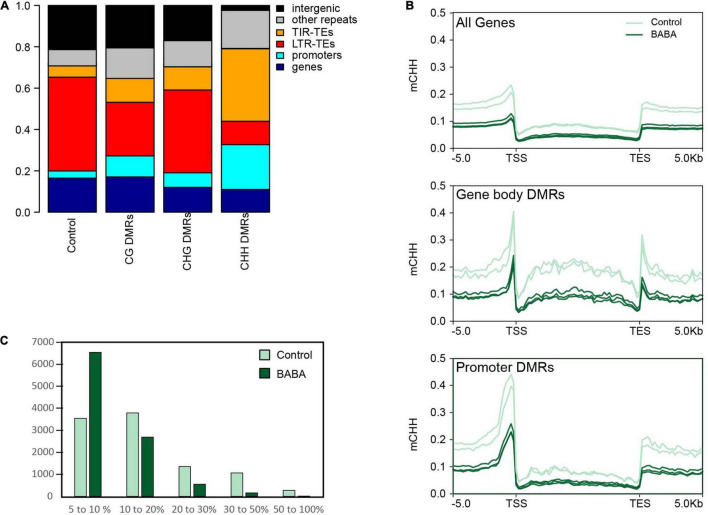
Distribution patterns of altered methylation. **(A)** Stacked bar plot displaying the proportions of BABA-induced DMRs overlapping different genomic features on the tomato genome. Data are shown for CG DMRs (*n* = 14,345), CHG DMRs (*n* = 24,941) and CHH DMRs (*n* = 11,709) and compared with the overlap found for a control set of 10,000 randomly selected regions of 200 bp. Promoters are defined as the DNA sequence located 1 kb upstream of the transcription start site of each annotated gene. **(B)** Averaged CHH methylation profiles across standardised tomato gene models for all tomato genes (*n* = 35,768) and genes overlapping DMRs in their gene body (*n* = 1,152) or in their promoter (*n* = 2,865). Plots show proportions of methylated cytosines for control (pale green) and BABA primed (dark green) methylomes. **(C)** Frequency histogram showing numbers of genes with different levels of total CHH methylation (%) in control and BABA-treated samples.

To investigate whether cytosine hypomethylation occurs at specific genomic regions, we calculated the portion of DMRs overlapping annotated features on the tomato reference genome in comparison to a set of randomly generated regions (Figure 2A). We observed that CHH DMRs tended to be highly enriched in Class II TEs with terminal inverted repeats (TIRs) and at the promoters of genes (defined as a 1 kb DNA sequence upstream of the transcription start), while they are under-represented in intergenic regions and Class I LTR retrotransposons (Figure 2A). Remarkably, the DMR enrichment correlates well with the chromosomal distribution of TE families in tomato. Indeed, LTR-TEs are mostly located in the central body of the chromosome where we identified relatively few DMRs, whereas TIR-TEs are more abundant on chromosome arms (Supplementary Figure 3A), where most hypo-DMRs were found. Nonetheless, the identified CHH DMRs appeared to be similarly enriched in each of the major TIR-TE superfamilies (Supplementary Figure 3B), which each display a similar decrease in CHH methylation in BABA treated samples in proportion to their methylation in control plants (Supplementary Figure 3C). Taken together, these results suggest that whilst BABA affects methylation in tomato in all cytosine contexts, only the CHH context displays an essentially unidirectional change (hypomethylation; Figure 1B) and a specific distribution (Figure 2A), suggesting a potential direct association with BABA-induced resistance.

### Defence Genes Are Targets of CHH Hypomethylation

To investigate the possibility that CHH hypomethylation might contribute to the priming of induced resistance responses, we identified genes that overlap with CHH DMRs in their transcribed region or in their promoter region. We found a total of 1,152 and 2,865 genes overlapping CHH DMRs in their transcribed region or in their promoter, respectively (Supplementary Table 2). We then compared the averaged CHH methylation profile of all tomato genes against the profile of genes overlapping CHH DMRs. We observed that in control plants, genes with CHH DMRs tend to be more heavily methylated relative to the average in tomato, but that in BABA-treated plants methylation is reduced to levels closer to the average (Figure 2B). We also analysed CHH methylation for all annotated genes independently of the presence of DMRs. We observed a much lower frequency of highly methylated genes and increased proportion of weakly methylated genes in BABA-treated samples relative to mock-treated samples (Figure 2C). Together, these results indicate that BABA treatment decreases CHH methylation mostly at highly methylated genes. To investigate the types of genes affected in CHH methylation by BABA treatment, we performed GO term enrichment analysis on the set of genes for which there were CHH DMRs overlapping either the promoter or transcribed region. Interestingly, GO terms significantly enriched in this set of genes included terms associated with stress responses, such as oxidative stress responses and cell wall biosynthesis, and terms associated with regulatory processes, e.g., “protein phosphorylation” and “regulation of cellular process” (Table 1). This suggests that BABA might contribute to the priming of defence responses by changing the epigenetic regulation of a specific set of stress responsive genes.

**TABLE 1:** GO terms from the biological process ontology significantly over-represented amongst genes with CHH DMRs overlapping either the promoter or transcribed region.

GO biological process*	Fold Enrichment	FDR
Pectin catabolic process	2.67	3.1 × 10^–2^
Response to oxidative stress	2.13	5.4 × 10^–3^
Antibiotic metabolic process	2.04	2.7 × 10^–2^
Sulphur compound metabolic process	2.01	8.6 × 10^–3^
Cellular oxidant detoxification	1.89	2.8 × 10^–2^
Secondary metabolic process	1.8	3.2 × 10^–2^
Inorganic cation transmembrane transport	1.76	5.9 × 10^–3^
Carboxylic acid biosynthetic process	1.74	1.1 × 10^–2^
Cofactor metabolic process	1.74	3.0 × 10^–3^
Cellular amino acid metabolic process	1.7	3.8 × 10^–2^
Peptide transport	1.62	1.5 × 10^–2^
Cellular lipid metabolic process	1.58	4.1 × 10^–2^
Macromolecule localisation	1.5	3.8 × 10^–2^
Oxidation-reduction process	1.45	7.7 × 10^–6^
Protein phosphorylation	1.39	1.3 × 10^–2^
Nucleic acid metabolic process	1.2	4.8 × 10^–2^
Regulation of cellular process	1.19	3.9 × 10^–2^

Data show the enrichment in the list of genes with DMRs relative to all annotated genes. FDR; P-value corrected for false discovery rate. *Where multiple related terms were identified as significant, only the highest-level term (i.e., deepest branch) in the relevant GO hierarchy is shown.

**FIGURE 3:**
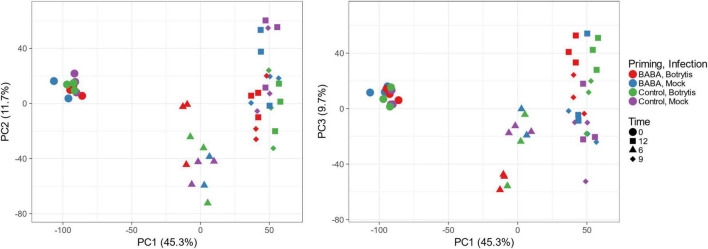
Principal component analysis of transcriptomic responses to *Botrytis cinerea* infection in leaves of control and BABA-primed tomato plants. PCA was performed using the Affymetrix signals from the 7767 probe sets remaining after initial filtering. Each data point represents a single RNA sample, collected from a pool of 16 inoculation sites from two leaves each from two independent plants. Colours are used to identify treatment and the different shapes indicate time points after inoculation.

To pursue the link between differential methylation and priming in more depth, we used a transcriptomic approach to identify genes that showed primed expression during pathogen infection following BABA treatment. We used the Affymetrix tomato genome array to profile expression of genes in plants inoculated with spores of the fungal pathogen *Botrytis cinerea*, an important pathogen of tomato and many other horticultural crops. Disease lesion measurements performed on plants used in the same experiment confirmed primed resistance against *B. cinerea* infection (Supplementary Figure 1C). After data normalisation and filtering, we applied a principal component analysis (PCA) to provide an overall picture of the impact of priming and infection across the full data set. The largest source of variation, represented by PC1, was time, whilst PCs 2 and 3 represent treatment effects. Plots of PC2 and PC3 against PC1 clearly reveal the primed transcriptional response to infection (Figure 3). At *t* = 0, all samples cluster together, indicating similar patterns of gene expression in all treatment groups and therefore no substantial sustained transcriptional response to the original BABA treatment. At 6, 9, and 12 h after inoculation, there is separation of the data along PC2 and PC3 that reflects the impact of *Botrytis* infection. However, at 6 h, the response to infection is much more apparent for the BABA-treated plants, which tend to separate clearly from the mock-inoculated leaves, whilst infection has a much smaller impact on leaves from control plants. This suggests that priming leads to a more rapid response to pathogen infection (Figure 3).

**FIGURE 4:**
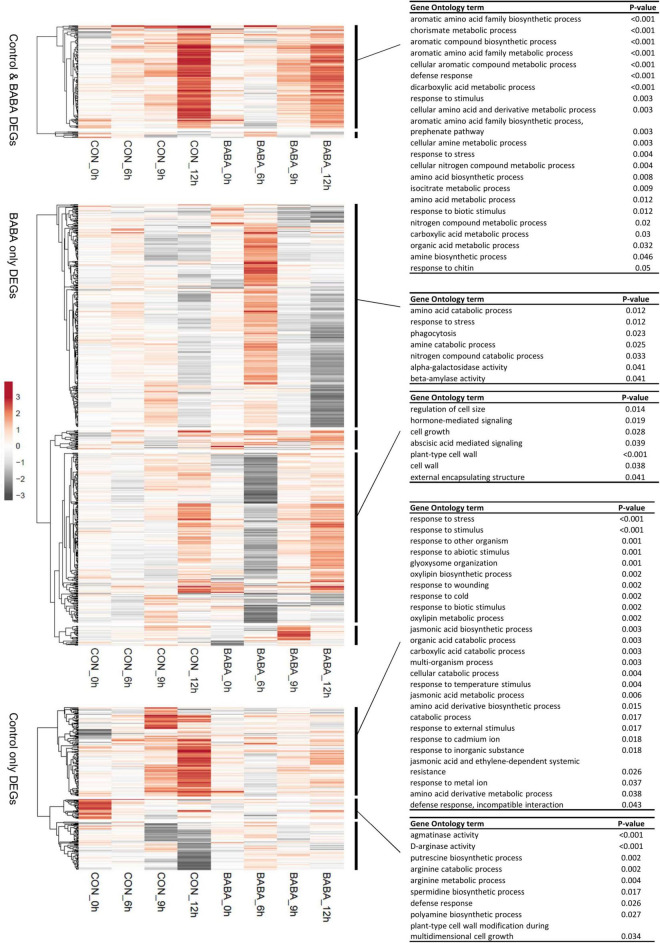
Expression profiles of genes exhibiting differential expression in response to *Botrytis cinerea* infection. Hierarchical clustering was used to separately profile genes from three main categories (those differentially expressed only in control plants, only in BABA-primed plants, or in both) Clustering was performed based on log2 fold-change values (*Botrytis*-inoculated relative to mock-inoculated leaves) at each time point (0, 6, 9, and 12 h post-inoculation) in control (CON) and BABA-treated leaves. Clustering of genes (rows) is based on correlation distance clustering and average linkage. Unit variance scaling was applied to rows, but values are not centred. Grey shading represents the degree of down-regulation (negative log_2_ fold-change values) in infected tissues of a gene at that particular time point, whilst up-regulation (positive log_2_ fold-change values) are shown in orange, and white indicates no difference in transcript levels between mock and pathogen-infected leaves. Functional profiling of the major clusters was performed using GO term enrichment analysis. Where identified, statistically significant GO terms are listed on the right of the figure.

Genes differentially expressed between mock- and pathogen-inoculated leaves were identified using a statistical method that considers the full time course to identify differentially expressed genes (DEGs). Using this approach to identify pathogen responsive DEGs separately from control and primed plants, we found 445 DEGs responding to infection in control plants and 935 from BABA treated plants (Supplementary Tables 3A,B), suggesting that not only was the response to infection earlier in primed plants, but also more extensive. Of the DEGs identified by this analysis, 188 were common to both control and BABA treatment groups, leaving 257 DEGs unique to control plants and 747 unique to primed plants (Supplementary Table 3C). Each of these three main groups of DEGs was classified by hierarchical clustering of the responses to infection (fold-change values) of control plants and primed plants at each time point. Then, co-expressed genes in each group were analysed by GO term enrichment (Figure 4). The group of common DEGs was comprised mainly of genes upregulated at 12 h after infection with similar levels in control and BABA plants. These genes were enriched in GO terms associated with amino acid metabolism, carboxylic acid metabolism and general stress responses. The DEGs unique to the BABA treated plants are candidates for primed genes, and comparisons of their expression patterns confirmed for the large majority of these genes, that whilst patterns of expression across the time course of infection were similar for both control and primed plants, the magnitude of the transcriptional response was greater in the primed plants. Notable GO terms associated with one major cluster from this category relate to abscisic acid signalling and cell walls, both of which are key elements of resistance to *B. cinerea* (Asselbergh et al., 2007; Vicedo et al., 2009). GO terms linked with stress responses and stress-related hormone signalling were also enriched in the list of genes responding to infection only in control plants.

**FIGURE 5:**
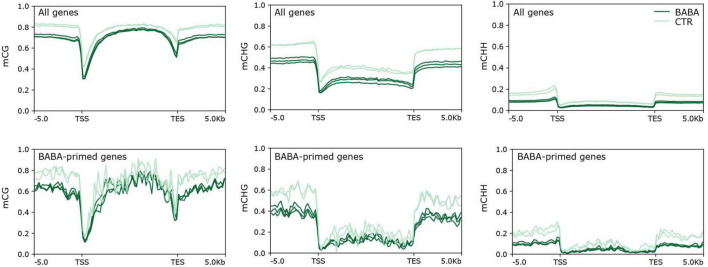
Methylation density alignments for mCG, mCHG and mCHH methylation across standardised profiles for all tomato genes, and for *Botrytis*-responsive, BABA-primed genes. X-axes show 5 kb distances upstream of the transcription start site (TSS) and transcription end site (TES) of the standardised genes. Plots show proportions of methylated cytosines for control (pale green) and BABA primed (dark green) methylomes.

We next considered whether genes primed by BABA for a stronger response to *B. cinerea* infection might also be targets for altered cytosine methylation. To this end, we identified genes that overlap with CG, CHG and CHH DMRs in their transcribed region or in their promoter region. Sites representing gain and loss of methylation were considered separately for CG and CHG contexts, for which both hyper- and hypoDMRs had been identified (Figure 1B). Lists of genes overlapping hyper- and hypoDMRs in each sequence context are provided in Supplementary Table 2. We next compared the frequency of genes containing DMRs in the lists of DEGs with their frequency in the whole genome (Table 2). Genes with CG or CHG DMRs were generally under-represented or showed no enrichment in the lists of DEGs in all three categories (control only, control and BABA, BABA only). Under-representation of CG and CHG DMRs was especially noticeable for DEGs identified only in control plants (i.e., in the absence of BABA), although with one exception, the degree of under-representation of CG and CHG DMRs was generally not statistically significant. In contrast, CHH hypoDMRs were significantly enriched in the promoters of all DEGs and in the gene body of those DEGs unique to BABA-treated plants (Table 2). Despite the significant enrichment of CHH DMRs in DEGs, the proportion of DEGs with DMRs was still relatively small (∼5% in gene bodies and 15% in promoters), and alignments of annotated gene features did not identify any obvious differences between the overall methylation pattern of primed genes and the pattern seen in the whole genome for any of the three sequence contexts (Figure 5).

**TABLE 2:** Representation of DMRs amongst the three main categories of DEGs.

	CG Gain		CG Loss		CHG Gain		CHG Loss		CHH Loss	
	Gene	Promoter	Gene	Promoter	Gene	Promoter	Gene	Promoter	Gene	Promoter
Control only	0.45	0.26	0.78	0.67	0.50	0.00	0.39*	0.87	1.72	1.55*
BABA and control	0.30	0.34	0.51	1.46	0.66	0.87	0.52	1.03	1.50	1.89**
BABA only	1.01	1.24	0.68	1.15	0.61	0.92	0.98	1.12	1.64**	1.72***

Figures show the enrichment of DMRs in DEG lists (proportion of genes in the DEG list containing DMRs relative to the proportion of genes with DMRs in the whole genome). Enrichment was calculated separately for hypermethylation (gain) and hypomethylation (loss) in CG, CHG, and CHH methylation contexts in the transcribed region (Gene) and promoter region of each gene. Asterisks represent statistical significance (*P < 0.05, **P < 0.01, ***P < 0.001 by Fisher’s exact test).

## Discussion

A wide variety of biotic stressors and chemical treatments, including BABA, can trigger priming of IR. Under the appropriate conditions, such priming effects can be long-lasting. Here, we show that root drenching tomato plants at the seedling stage with 0.5 mM BABA provides long-lasting priming of both SA- and JA-dependent resistance, effective against *P. syringae* and *B. cinerea* respectively. This treatment was chosen on the basis of previous dose-response experiments that indicated that inhibition of growth of 2- to 3-week-old plants becomes significant at concentrations of 1 mM and above (Luna et al., 2016). Although BABA caused a temporary reduction in seedling growth, control and primed plants were morphologically similar at the time we measured defence responses and DNA methylation profiles. Transcriptome analysis following inoculation with *B. cinerea* was consistent with a typical pattern of priming for an enhanced future stress response. There was no sustained effect of BABA treatment on gene expression (expression profiles were similar in control and primed plants prior to inoculation), but root drenching of seedlings led to an earlier and stronger transcriptional response to subsequent infection (Figures 3, 4). Functional classification of genes forming co-expressed clusters based on transcriptional responses to *B. cinerea* infection revealed many expected processes, such as stress-related hormone biosynthesis and signalling, secondary metabolism, cell wall metabolism and other general stress response categories. In addition, numerous GO terms related to amino acid and carboxylic acid metabolism were enriched. These observations are consistent with previous transcriptomic and metabolomic surveys of similar priming responses (Bengtsson et al., 2014; Finiti et al., 2014; Camañes et al., 2015). Whilst we identified no major differences in gene expression between controls and BABA-primed plants before inoculation, WGBS of uninfected plants revealed substantial differences in DNA methylation between these two groups, and in particular, hypomethylation at CHH positions in the chromosome arms.

### Effects of Environmental Stress on DNA Methylation

Various studies over recent years have used whole genome methylation sequencing approaches to investigate the role of DNA methylation in plant responses to environmental stress, particularly in the context of long-term epigenetic stress memory and priming (e.g., Wibowo et al., 2016; Colicchio et al., 2018; Stassen et al., 2018; Song et al., 2020; Wang et al., 2021). These and other similar studies typically find dynamic responses, including frequent observations of CHH hypomethylation following stress. For example, decreased DNA methylation has been observed following infection by bacterial and fungal pathogens (Pavet et al., 2006; Dowen et al., 2012; Geng et al., 2019) and by plant parasitic nematodes (Hewezi et al., 2017; Atighi et al., 2020). In terms of abiotic stress, a net reduction in methylation also occurred following cold stress in Tartary buckwheat (Song et al., 2020), whereas methylation levels increased following exposure to salinity stress in Arabidopsis and rice (Wibowo et al., 2016; Wang et al., 2021). In some cases, differentially methylated regions (DMRs) have been suggested to co-localise with stress responsive genes, suggesting direct regulation of expression by DNA methylation (Colicchio et al., 2018; Atighi et al., 2020; Song et al., 2020; Wang et al., 2021). In contrast, other studies failed to establish direct links between differential methylation and gene expression, suggesting instead that where changes in DNA methylation result in stress-induced changes in transcriptional activity, this occurs via *in trans* regulatory interactions, in which the methylation status at one or more chromosomal loci has long-range effects on other regions of the genome (López Sánchez et al., 2016; Cambiagno et al., 2018; Stassen et al., 2018; Xu et al., 2018; Furci et al., 2019).

Leaves of tomato plants treated with BABA carried a clear epigenetic signature of priming 7 days following treatment, consisting of a significant global reduction in cytosine methylation in all three sequence contexts. This is consistent with observations of Kuźnicki et al. (2019), who, using immunological approaches, detected a general reduction in global methylation upon BABA priming of potato plants. These authors also found rapid changes in the expression of genes encoding enzymes involved in both DNA methylation (*CHROMOMETHYLASE 3* and *DOMAINS REARRANGED METHYLTRANSFERASE 2*) and demethylation (*DEMETER-LIKE DNA DEMETHYLASE 2* and *REPRESSOR OF SILENCING 1*) in response to BABA treatment. In our experiments with primed tomato leaves, the close to 50% reduction in the frequency of mCHH in the genome was particularly striking (Figure 1A), and whilst the net reduction in CG and CHG methylation reflected a balance of both hyper- and hypomethylation (as determined by identification of DMRs), almost all CHH DMRs were hypomethylated. Furthermore, hypo-DMRs in all three contexts were concentrated in the chromosome arms, where the majority of genes and DNA TEs are localised, rather than in the pericentromeric regions which are most heavily methylated. This suggests that in our experiments, a targeted reduction of methylation, especially at CHH positions, occurred in gene-rich regions of tomato chromosomes in response to BABA priming. Interestingly, this pattern closely resembles the DNA methylation changes in nematode feeding galls in rice observed by Atighi et al. (2020). Nematode parasitism caused a global reduction in methylation, with the biggest changes seen for mCHH. Almost all (99.97%) CHH DMRs were hypomethylated, and enriched in gene promoters and in TEs from the DNA and retroelement short interspersed nuclear element families (Atighi et al., 2020). Interestingly, a similar distribution of CHH hypomethylation is observed in tomato mutants deficient in RNA-dependent DNA methylation (RdDM; Gouil and Baulcombe, 2016), suggesting that BABA may influence the methylome at least in part via reduced RdDM activity.

### Mechanisms for Priming of Stress Responses by DNA Hypomethylation

Despite the similarity of the effect of priming on the tomato methylome to responses seen following various other biotic stresses, we did not detect a widespread correspondence between differential methylation and differential gene expression. There was no enrichment of CG or CHG DMRs in genes differentially regulated by pathogen infection. However, consistent with studies discussed above, there was a significant enrichment in genes overlapping CHH hypoDMRs in our DEG lists (Table 2). Nevertheless, the overall enrichment was relatively low (less than twofold) and the total proportion of DEGs containing CHH DMRs in their promoters was only around 15% (compared with 8% across the entire genome). The majority of pathogen-responsive genes therefore do not contain DMRs (as defined under the parameters applied in our analysis). Furthermore, enrichment of genes with DMRs was similar for DEG lists identified from both control and BABA-treated plants. This suggests that either targetting of DMRs to stress responsive genes is not causally linked with transcriptional priming, or alternatively that our criteria for defining a primed gene are ineffective. For example, genes in the “control only” and “control and BABA” categories that we considered not to be primed, might exhibit enhanced expression in BABA treated leaves at time points other than those sampled here. Regardless, we did not detect differential methylation in the majority of genes exhibiting primed expression. It is likely, therefore, that the changes in DNA methylation associated with BABA treatment are predominantly linked indirectly to the transcriptional response of primed plants challenged with the pathogen. Nevertheless, the set of genes overlapping CHH DMRs was enriched for several GO terms linked with stress responses, especially in relation to oxidative stress (Table 1). Since oxidative stress is common to many biotic and abiotic stresses, the preferential CHH hypomethylation of genes linked with oxidative stress may be related to the ability of BABA to confer resistance or tolerance against a wide range of different stressors (Baccelli and Mauch-Mani, 2016; Cohen et al., 2016). Indeed, improved oxidative stress tolerance is commonly reported as a mechanism for BABA priming (e.g., Dubreuil-Maurizi et al., 2010; Pastor et al., 2013; Roylawar and Kamble, 2017). In addition, GO terms linked to general regulation were also over-represented amongst genes with CHH DMRs, which may be one mechanism for the *in trans* regulation of responses to infection by (de)methylation identified by López Sánchez et al. (2016) and Stassen et al. (2018).

In addition to the presence of CHH DMRs in the promoters and bodies of tomato genes, CHH DMRs were also highly enriched in Class II TEs, i.e., DNA transposons with TIRs. In contrast, TEs were relatively unaffected by BABA treatment at mCG positions. In tomato, TIR TEs are preferentially localised in the chromosome arms along with protein-coding genes, supporting the conclusion that BABA priming is marked by hypomethylation in the chromosome arms. Activation of TEs resulting from demethylation is a well-known response to environmental stress, and where such TEs exist close to protein-coding genes, the impact of their demethylation can extend to an influence on the expression of those genes (Negi et al., 2016; Horváth et al., 2017; Zhang et al., 2018). Several previous studies have identified priming and/or elevated transcriptional activity of stress responsive genes as a consequence of their proximity to demethylated TEs, and particularly DNA TEs (Dowen et al., 2012; Wibowo et al., 2016; Geng et al., 2019; Wang et al., 2021). Based on the patterns of changes in methylation and gene expression identified in our experiments, we suggest that priming of stress responses by BABA likely arises from a combination of direct changes in methylation at loci encoding genes that either have direct defence functions or which play regulatory roles during stress, the influence of hypomethylation at loci containing TEs on adjacent defence genes, and *in trans* regulatory effects that operate over longer distances.

### Specificity of Priming Memory

One of the most intriguing properties of BABA is the wide range of different stresses against which it confers protection, including infection by both biotrophic and necrotrophic pathogens. Biotrophic pathogen infection is typically countered by SA-dependent IR, whilst necrotrophic infection is defended via JA-IR. These two principal defence pathways are antagonistic under most circumstances; a property that is maintained by long-term priming such that priming for an enhanced capability of one pathway comes at a cost of reduced activity of the other (Luna et al., 2012; Singh et al., 2017; López Sánchez et al., 2021). This is also true where epigenetic modifications of the genome result in altered defence responses. For example, mutants in *DNA-DIRECTED POL V SUBUNIT 1* (also known as *NRPE1*), which is involved in RNA-directed DNA methylation (RdDM), display CHH hypomethylation and are resistant to biotrophic pathogens as a consequence of being primed for SA-IR, but are more susceptible to necrotrophic fungi (López et al., 2011; López Sánchez et al., 2016). Despite this well-conserved antagonism, BABA nevertheless primes both SA- and JA-dependent pathways, as demonstrated here and by numerous other studies (Baccelli and Mauch-Mani, 2016; Cohen et al., 2016; Luna et al., 2020). There is therefore an apparent contradiction between the concept of an association between hypomethylation and priming of both SA-IR and BABA-IR. However, CHH hypomethylation is by no means exclusively linked with activation of SA-IR and consequent reduced activity of alternative stress resistance pathways. For example, several hypomethylated loci that conferred biotrophic pathogen resistance were identified in a screen of recombinant inbred lines derived from the hypomethylated *decreased DNA methylation1-2* (*ddm1-2*) Arabidopsis mutant. Each of these epiQTLs conferred resistance via primed SA-IR as expected, but also promoted SA-independent deposition of callose at sites of fungal penetration (Furci et al., 2019). Furthermore, salinity tolerance and JA-dependent resistance to necrotrophs were not affected (or were even increased) in these lines. Meanwhile, CHH hypomethylation resulting from polyploidy in rice resulted in primed JA responses and enhanced salinity tolerance (Wang et al., 2021). Together, these observations suggest that CHH hypomethylation can provide a generic mechanism for priming for stress resistance, but that the resistance pathways that are affected depend on the initial stimulus that leads to the loss of CHH methylation, perhaps via the stimulus-specific generation of siRNAs that control CHH methylation at relevant genetic loci.

## Conclusion

Our data suggest a model in which the BABA seedling root drench treatment leads to remodeling of the tomato methylome, with major changes targeted toward the gene-rich chromosome arms. Differential methylation arises in all three sequence contexts, but CHH DMRs show a striking bias toward hypomethylation and are enriched in gene promoters and Class II TEs. The presence of CHH hypoDMRs is proportionately higher in the promoters of genes showing stress-responsive transcriptional activation, but many genes that demonstrate recognisable patterns of priming do not contain DMRs. We therefore conclude that CHH hypomethylation is a key feature of priming by BABA, and that it might be linked to enhanced stress resistance phenotypes through several mechanisms. Firstly, transcriptional priming of stress-responsive genes may occur through loss of CHH methylation in their own promoters or gene bodies, or via primed activity of their transcriptional regulators caused by CHH hypomethylation in those genes. Secondly, there are also likely to be more indirect mechanisms impacting more widely on local chromosome architecture, either through local effects on chromatin accessibility caused by changes in TE methylation, or by altered topology of chromosome packing in the nucleus. Testing the validity of these hypotheses remains a key challenge in our understanding of long-term stress memory in plants.

## Data Availability Statement

The datasets presented in this study can be found in online repositories. The names of the repository/repositories and accession number(s) can be found below: https://www.ncbi.nlm.nih.gov/geo/, GSE190782.

## Author Contributions

MR, JT, EL, and RA-V conceived the study. RA-V, DW, and GH performed the experiments. MR, MC, RA-V, AB, DW, and GH analysed the data. MR and MC wrote the manuscript with contributions from RA-V, AB, EL, and JT. All authors contributed to the article and approved the submitted version.

## Conflict of Interest

The authors declare that the research was conducted in the absence of any commercial or financial relationships that could be construed as a potential conflict of interest.

## Publisher’s Note

All claims expressed in this article are solely those of the authors and do not necessarily represent those of their affiliated organizations, or those of the publisher, the editors and the reviewers. Any product that may be evaluated in this article, or claim that may be made by its manufacturer, is not guaranteed or endorsed by the publisher.

## Footnotes

1
https://solgenomics.net

2
http://ted.bti.cornell.edu/
