# Supplementary material for: Long-Lasting Defence Priming by β-Aminobutyric Acid in Tomato Is Marked by Genome-Wide Changes in DNA Methylation
Source: Front Plant Sci. 2022 Apr 15;13:836326. doi: 10.3389/fpls.2022.836326 (PMC9051511; doi:10.3389/fpls.2022.836326)
Supplement: Supplementary file 1 [file Data_Sheet_2.pdf]

Supplementary Material

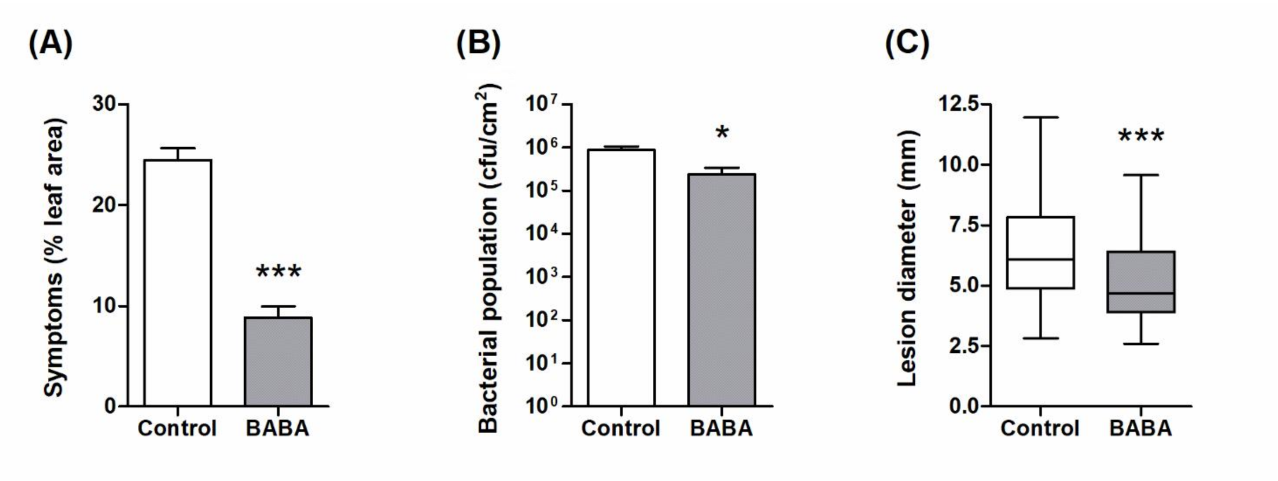

Supplementary Figure 1. Pathogen resistance in tomato primed by BABA seedling root drench. *Pseudomonas syringae* resistance in control and primed plants used for WGBS was measured in terms of visible symptoms and bacterial populations in infected leaves. (**A**) Symptoms (area of chlorosis and necrosis) assessed 10 days after pathogen infection in tomato plants inoculated with *P. syringae* after BABA treatment or water (Control). ****P*= 0.0006 by Student's t-test (n=3; F test to compare variances: F=1.078, DFn=2, Dfd=2; *P*=0.96) (**B**) Colony forming units (cfu) in tomato plants six days after infection. **P*= 0.04 by Student's t-test (n=3; F test to compare variances: F=3.613, DFn=2, Dfd=2; *P*=0.43) (**C**) *Botrytis cinerea* resistance in control and primed plants used for microarray analysis. Plots show median and quartile distributions of lesion diameters. ****P*= 0.0005 (n=76) by Mann Whitney test following D'Agostino & Pearson omnibus normality test.

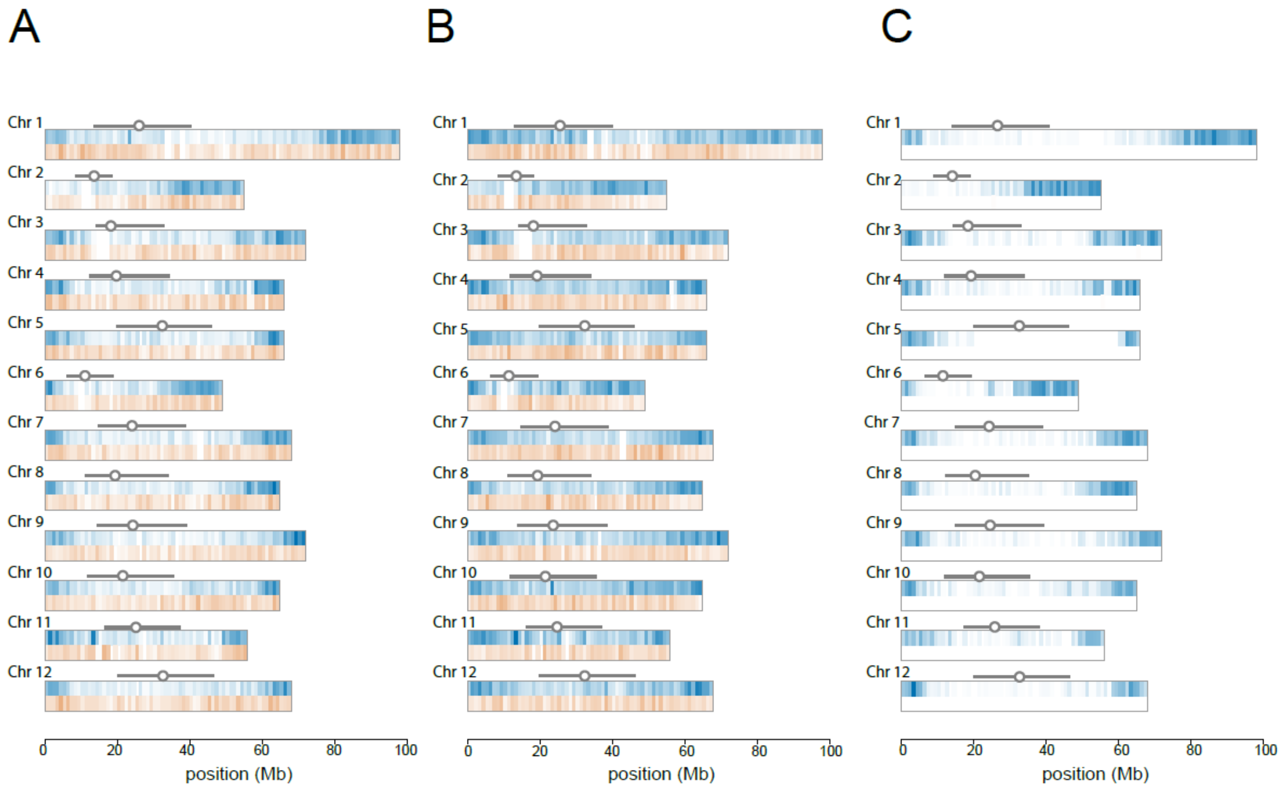

Supplementary Figure 2. Distribution of DMRs in response to BABA treatment in tomato chromosomes, plotted with *DMRCaller* in 1 Mb windows for (**A**) mCG, (**B**) mCHG, and (**C**) mCHH contexts. Hypo-DMRs and Hyper-DMRs are displayed in blue and red respectively. The intensity of the colour is proportional to the density of DMRs. The positions of heterochromatic regions (grey lines) and centromeres (white circles) as defined by Sato *et al*., 2012, are displayed above each chromosome.

Sato, S., Tabata, S., Hirakawa, H., Asamizu, E., Shirasawa, K., Isobe, S., et al. (2012). The tomato genome sequence provides insights into fleshy fruit evolution. *Nature* 485, 635–641. doi.: 10.1038/nature11119.

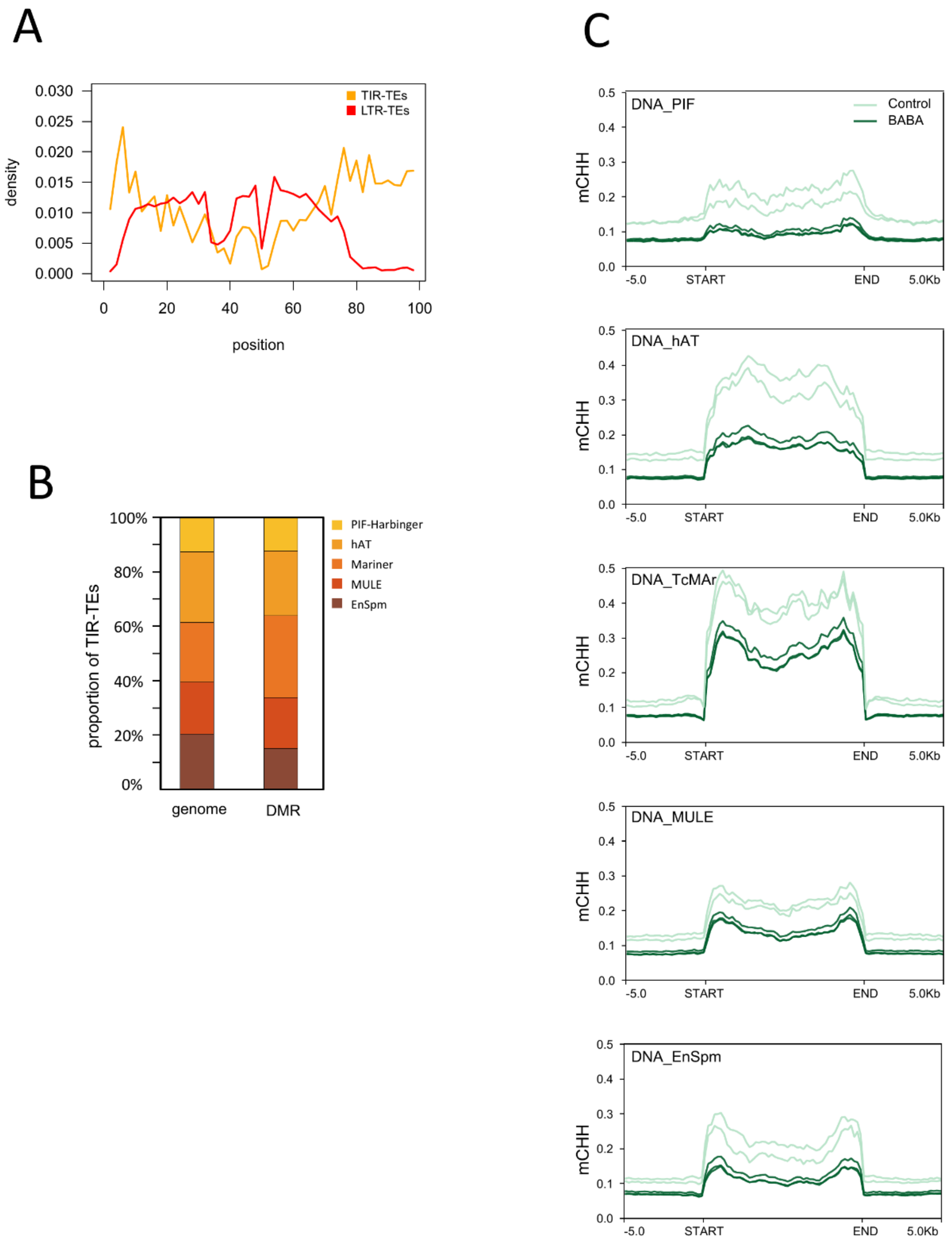

Supplementary Figure 3. Occurrence of BABA-induced DMRs in tomato transposable elements. (**A**) Graph showing the density of annotated TIR-TEs and LTR-TEs along chromosome 1 of the tomato reference genome. (**B**) Proportion of TEs belonging to the main TIR-TE superfamilies in the tomato genome (genome) and for TIR-TEs overlapping DMRs (DMR). (**C**) Averaged CHH methylation profiles across standardised TEs belonging to the main five TIR-TE superfamilies in tomato. Plots show proportions of methylated cytosines for control (pale green) and BABA primed (dark green) methylomes.
